# Nonverbal Auditory Cues Allow Relationship Quality to be Inferred During Conversations

**DOI:** 10.1007/s10919-021-00386-y

**Published:** 2021-10-22

**Authors:** R. I. M. Dunbar, Juan-Pablo Robledo, Ignacio Tamarit, Ian Cross, Emma Smith

**Affiliations:** 1 Department of Experimental Psychology, University of Oxford, Radcliffe Observatory Quarter, Anna Watts Building, Oxford, OX2 6GG UK; 2 Centre for Music and Science, Faculty of Music, University of Cambridge, 11 West Road, Cambridge, CB3 9DP UK; 3 Laboratoire Interpsy, Campus Lettres et Sciences Humaines et Sociales, Université de Lorraine, 23, Bd Albert 1er, 54015 Nancy cedex, France; 4 Grupo Interdisciplinar de Sistemas Complejos, Departamento de Matemáticas, Universidad Carlos III de Madrid, 28911 Leganés, Madrid, Spain; 5 Wysing Arts Centre, Fox Road, Bourn, Cambridge, CB23 2TX UK; 6 Millennium Institute for Caregiving Research (MICARE), Santiago, Chile

**Keywords:** Conversation, Nonverbal cues, Verbal content, Relationship quality, Mehrabian’s Conjecture

## Abstract

The claim that nonverbal cues provide more information than the linguistic content of a conversational exchange (the Mehrabian Conjecture) has been widely cited and equally widely disputed, mainly on methodological grounds. Most studies that have tested the Conjecture have used individual words or short phrases spoken by actors imitating emotions. While cue recognition is certainly important, speech evolved to manage interactions and relationships rather than simple information exchange. In a cross-cultural design, we tested participants’ ability to identify the quality of the interaction (rapport) in naturalistic third party conversations in their own and a less familiar language, using full auditory content versus audio clips whose verbal content has been digitally altered to differing extents. We found that, using nonverbal content alone, people are 75–90% as accurate as they are with full audio cues in identifying positive vs negative relationships, and 45–53% as accurate in identifying eight different relationship types. The results broadly support Mehrabian’s claim that a significant amount of information about others’ social relationships is conveyed in the nonverbal component of speech.

**Supplementary Information:**

The online version contains supplementary material available at 10.1007/s10919-021-00386-y.

Language has been the single most important evolutionary development achieved by humans, and, aside from its obviously central role in culture, much has been made of its value as a medium through which information can be transmitted and cooperation negotiated (Tomasello, 2010). Within this, there has been a longstanding interest in whether the verbal or nonverbal aspects of speech carry more weight (Argyle et al., 1970, 1971; Burgoon et al., 2002; Manusov & Trees, 2002; Mehrabian & Ferris, 1967; Mehrabian & Wiener, 1967). Early claims were made for the pre-eminence of nonverbal cues (the Mehrabian Conjecture), at least with respect to the communication of affect, with more than 90% of the information in conversation-based exchanges conveyed by nonverbal cues like intonation, phrasing, and facial expressions (Mehrabian, 1972). Mehrabian’s claim has, however, been questioned, either on grounds of the ecological validity of the experimental tasks (Allen & Atkinson, 1981) or because the experimental designs suffered from methodological (Furnham et al., 1981; Trimboli & Walker, 1987), statistical (Hegstrom, 1979), or contextual (Grahe & Bernieri, 1999; Lapakko, 1997) confounds. That said, no one would doubt that nonverbal cues provide a great deal of information during verbal exchanges: indeed, they are what allow us to infer the meaning of an utterance. It is Mehrabian’s quantitative claim that only ~ 7% of the meaning of any utterance is encapsulated in the verbal component that seems to have attracted the most attention. Later studies that have tried to circumvent the methodological issues have yielded less extreme estimates.

More recently, there has been renewed interest in the topic, in most cases exploiting alternative paradigms. Summerfield (1979), for example, found that adding visual cues increased accuracy in a speech recognition task from 23 to 65%. Similarly, Mileva et al. (2018) found that both audio and visual channels independently inform judgments over characteristics like social dominance and trustworthiness, with different channels carrying different weights in different contexts. Using actors speaking a short phrase, Cowen et al. (2019) found that subjects were able to identify twelve different emotions from prosodic cues alone, and were able to do so across two different cultures. Similar results were obtained by Mixdorff et al. (2017) with a smaller range of emotions. Raters were, nonetheless, far from perfect: Cowen et al. (2019) found that, on average, only 25% of raters converged on the same emotion descriptor (against a chance level of 14%).

Perhaps the most serious criticism of previous studies is that they have focused on very low-level information transfer (recognition of emotional states or status cues such as dominance). This is tantamount to focusing on the bricks and mortar while ignoring the intricacies of the buildings for which these are being used. Grahe and Bernieri (1999), for example, comment that simply recognizing an emotional expression or affective disposition is not the same thing as recognizing the degree of *rapport* between two interacting individuals. Rapport differs from disposition or simple affect in that it is the property of a dyad or a group. Human sociality, like that of all primates, is based on the quality of *relationships*, not just the meaning of individual signals (Hinde, 1995). Similarly, Pearce et al. (2017) have pointed out that the human (and primate) social world is much more complex than that implied in most psychological and neuroscience approaches where the focus has been entirely on dyadic interactions and the recognition of affect. They argue that, at the very least, we should distinguish between three distinct levels of sociality: social predispositions, dyadic relationships, and social networks (where third-party relationships become important) (see also Dunbar, 2018).

One recent attempt to rise to this challenge found that hearing a short clip of two people laughing together was sufficient to allow the listener to predict whether the pair were friends or strangers (with an accuracy of 53–67% across 24 different cultures) (Bryant et al., 2016). Although this is only just above chance level (at 50%), these results suggest that it may be possible to infer something about the quality of a social interaction from nonverbal cues alone. Moreover, any design that uses actors or the attempts of untrained participants to imitate emotional content are subject to considerable effects due to differences in acting skills (Mixdorff et al., 2017).

Although the knowledge-based economy, with its basis in formal instruction, has undoubtedly introduced a large number of contexts where technical information exchange is the sole objective (and nonverbal cues likely to be of limited importance), social exchanges remain the primary contexts in which we converse. Samples of natural everyday conversations indicate that two-thirds or more of conversation time is devoted to the exchange of social information about the conversants or a third party, with only a relatively modest 10% devoted to technical information exchange (Dahmardeh & Dunbar, 2017; Dunbar et al., 1997; Wiessner, 2014). More importantly, perhaps, much of our information on the quality of the social relationships in our communities comes not from what others confide in us but what we observe by watching third parties interacting. In understanding both the origins of speech and usages to which spoken exchanges are put, it is these social contexts that are by far the most important.

Here, we explore this question in more detail using short audio clips of actual conversational exchanges between two individuals. Our study differs from most previous studies in three key respects. First, our focus is on the natural situation in which much of our information about third party relationships comes from observing and overhearing others talking to each other, reflecting the fact that we are embedded in social networks with many individuals with whom we do not necessarily interact frequently (Dunbar, 2020; Sutcliffe et al., 2012). In contrast, previous studies have all focused on how we interpret emotional information from a single speaker’s brief utterance. Second, whereas most previous studies have focused on the emotional cues in utterances, we here focus on how we interpret the quality of the relationship as a whole. Third, our use of natural conversations ensures that the stimuli we use are ecologically valid, relatively natural, do not include prosodic exaggerations of the kind that are conventionally introduced by actors, and are focused explicitly on auditory cues.

Like all previous studies, we use a binary design (friendly versus unfriendly). However, to provide more analytical power, we used clips that illustrate a range of registers (Agha, 1999, 2008) within this dichotomy. This gives us two levels of analysis: a simple coarse-grained division between positive and negative interactions (based on four clips illustrating a range of registers for each) and a finer-grained level focused on shades of quality in relationships (but with a single sample in each case). Because humans are highly visual in the way they interface with the world, and this is often involuntarily dominant (see Mileva et al., 2018), we explicitly focus on audio clips in order to remove any confounding effects due to visual cues such as facial expressions or gestures.

The present study aims to assess the extent to which semantic and prosodic information is required for listeners to identify the quality of the relationship between speakers. Participants heard three separate versions of the same audio clip: the original clip with all prosodic and verbal cues preserved; a version in which we preserved the prosodic cues in full but removed the verbal content by delexicalizing the clip so that the words could not be identified; and a version in which the audio stream was converted to pure pitch tones so that only pitch and rhythm are preserved. We include the pitch-only condition because fundamental frequency has been shown to be focal in shaping prosodic and hence sociolinguistic judgments (Greenberg et al., 2009; Hellbernd & Sammler, 2016; Yaeger-Dror, 2002). Finally, we sampled native English and Spanish speakers listening to clips in both languages so as to determine whether language familiarity had an effect. Our design compares performance on the audio clips from which the verbal content has been progressively stripped from the original full audio clip. We take performance on the full audio clip as baseline, and ask how well subjects can do when they lack verbal content.

We make three explicit predictions derived from the presumption that verbal content is paramount in the inferences we make about third-party relationships. If verbal content is paramount, we expect performance to be above chance only in the full audio condition (H1a), whereas if nonverbal cues play a significant role, performance will be above chance even when the verbal content is degraded (delexicalized and pitch-only conditions) (H1b). Second, if verbal cues are paramount, we expect performance to rank in the order: full audio >  > delexicalized = pitch only, where performance in the latter two cases should be minimal (H2a); whereas, if nonverbal cues also play a role, we expect the clips to rank in the order: full audio > delexicalized > pitch only (i.e., in order of declining information) (H2b). More generally, if verbal content is crucial, we expect participants to perform better when listening to their own language than to a language with which they are less familiar (H3). We do not seek to address the question as to what prosodic features allow subjects to correctly identify relationship quality. That is certainly a question of interest, but would require a very different experimental design. Our question is simply: can people do it at all? That question has never been asked in this context—because no previous studies have looked at *relationship quality* as the outcome measure (all have focused on questions like simple emotional cue recognition).

## Method

The study was conducted online in two locations: 199 native English speakers resident in the UK undertook the experiment in English on the panel provider Prolific (www.app.prolific.co), and 139 native Spanish speakers resident in Spain undertook the experiment in Spanish on the IBSEN panel (www.ibsen.eu). In the UK sample, 75% of participants were aged 18–30 years, and 65% were female; in the Spanish sample, 82.3% were aged 18–30 years and 60.0% were female (with two specifying “other” for gender). In each case, half the subjects heard audio clips in their own native language, and half in the other language. Of the UK participants, none rated themselves as fluent in Spanish and only 1% described themselves as sufficiently competent to hold a casual conversation in the language. Of the Spanish participants, 45% declared themselves to be fluent in English and a further 35% declared that they were competent enough to hold a casual conversation. Four participants were excluded from the UK sample because, despite the filtering for native English speakers, they declared their fluency in English to be poor.

We focused on the ways in which prosodic cues may be implicated in inferences about the quality of the relationship between two speakers, based on a simple positive versus negative dichotomy. The positive or negative characteristics of the conversations to be used locate them at the extremes of something like Arndt and Janney's (1991) schema for emotive communication, with low intensity, value-laden-ness and minimal assertiveness represented by interactions in phatic contexts (sensu Malinowski, 1923) that exhibit high degrees of cooperativeness, while at the opposite extremes would be those likely to be encountered in contexts of intemperate impoliteness (see Culpepper et al., 2003).

We use the term ‘register’ to refer, somewhat loosely, to variations in the style of interaction (the *social meaning*, or rapport). The term derives from sociolinguistics and related disciplines where it refers both to what is said and how it is said, as well as by and to whom, in what circumstances, and under what constraints (Agha, 1999, 2008). Definitions vary, but most acknowledge the importance of a wide range of contextual and specific features (Biber & Conrad, 2001) that include: the participants (their relationships and attitudes toward the communication), the setting (including factors such as the extent to which time and place are shared by the participants, and the level of formality), the channel of communication, the production and processing circumstances (e.g., amount of time available), the purpose of the communication, and the topic or subject matter.

A number of frameworks for defining register have been suggested. Hymes (see Duranti, 1985) proposed that register can be encompassed by a complex speech-event model based around the mnemonic SPEAKING (*Situation*, *Participants*, *Ends*, *Act sequence*, *Key*, *Instrumentalities*, *Norms*, *Genres*), while Arndt and Janney (1991) put forward a three-factor model based on the *intensity*, *value-ladenness* and *assertiveness* of the speech produced. These definitions differentiate between conversational registers that are patently cooperative and affiliative and those that are unambiguously antagonistic, although more fine-grained distinctions might be apparent. We follow Arndt and Janney (1991) and use the term here simply to differentiate between qualities of conversational interaction as reflecting the relationship between the speakers.

Building on these definitions, we identified four ‘registers’ within each of the two categories of positive (phatic) and negative relationships (Table 1; see also Supplementary Materials). The choice of four registers is arbitrary, and is intended only to ensure breadth and balance across the two categories and to provide a framework for approximate equivalence when selecting samples from two different languages.

**Table 1:** Eight types of relationships represented in the audio clips

Context\Affect	Agreement	Disagreement	Gossip	Provocation
Positive	Free agreement	Disagree with respect	Phatic communion	Friendly provocation
Negative	Enforced agreement	Disagreement without regard	Malicious gossip	Aggressive provocation

Note that, in classifying clips, researchers had the full video clips available to them, not just the audio clips

Friendly Interaction:

1) Free Agreement: Speakers are in agreement with one another (as indicated by smiles and head nods)

2) Difference of opinion with respect: Speakers have different opinions to one another but wish to retain a positive relationship (exchanges are polite, but clearly promote contrary views)

3) Phatic communion: Speakers are not really bothered about the topic of conversation but are just passing time together (conversation is casual, light and easy-going)

4) Friendly provocation: Speakers are winding each other up as a joke but do not intend to upset each other (e.g. teasing, banter, “ribbing”, “joshing”)

Agonistic interaction:

5) Enforced agreement: Speaker says they agree but it is obvious from their behavior that they are not (agreement utterances are short and non-committal, and somewhat strained)

6) Disagreement without regard: Speakers have a difference of opinion and do not care if they upset / insult the other (they verge on, may even descend into, shouting matches)

7) Malicious gossip: Talking maliciously about someone who is not present

8) Aggressive provocation: Intentionally and maliciously trying to upset or provoke the other person

We should emphasize that we are not here testing the validity of this way of describing interactions. Rather, we are simply using it as a device to ensure that we have as wide a social range of stimuli as possible. This ensures that, rather than having eight clips of the same type of interaction, we are forced to introduce some variety into the selection of the clips. Since we use clips from two different language communities (English and Spanish), this also provides a framework that helps us to choose clips that are roughly comparable in sentiment. Our selections were based on full video clips with full visual content, giving us privileged information through an additional channel when choosing clips to use. The video samples we used are publicly available (mainly via YouTube).

Some studies (e.g., Sikveland & Zeitlyn, 2017; Zeller & Ogden, 2014) have used telephone conversational corpora (e.g., from radio phone-ins or call center recordings). However, these invariably involve one-off conversations between complete strangers. More importantly, conversations that lack a video channel do not have the same social quality as face-to-face interactions (Vlahovic et al., 2012), and as a result are more prone to ‘flaming’ and exaggerated responses. For this reason, we insisted on recordings explicitly made in social contexts (all participants in clear face-to-face visual contact) where the speakers were not strangers (i.e., were not negotiating novel relationships). We selected segments where there were only two speakers, even though there were more onlookers present (and who might have contributed to the conversation previously or subsequently to our excerpt). In our samples, there were 2–5 people present in the original clips, but always only two speakers.

The English samples were selected by ES, and the Spanish samples were then selected by JPRC (a native Chilean Spanish speaker) to be as close a match as possible to the English samples in terms of context and ambience. In both cases, the selections were made from the full video recordings, and benefited from the wider context in which the clips were embedded. Both sets of samples were subsequently independently assessed by two native English speakers (both authors) for the English clips and two native Spanish speakers (one an author) for the Spanish clips who had not been involved in either the choice of clips *or* the decision on which registers to use. The assessors undertook the same task as the experimental subjects (listening to each clip and deciding which of the eight registers described it best). The concordance between assessors was high and agreed with the original classifications.

The video clips were sourced from YouTube or existing databases (see Supplementary Materials Table S1). All were from live-recorded vlogs, lab-recorded conversations, reality TV shows (that were known to have minimal scripting), or live TV panel discussions, and all were in full video format. Clips averaged 25.2 ± 15.2 secs in duration, with no consistent difference between languages (unequal variances: *t*_8.7_ = 2.03, *p* = 0.074 2-tailed). In each case, there were two speakers (though in some cases there were other individuals present on screen). Because the clips derived from actual conversations, pacing, pitch, and emotional expression, as well as the interfacing of speaking turns, were relatively natural. Since our concern is specifically with audio communication, and the visual channel carries considerable relevant gestural information, we used only the audio tracks from the clips so as to avoid confounding the semantic and prosodic audio cues with information provided by facial expressions and gestures.

We used three versions of each clip as stimuli: (1) a pure audio version (the original audio track), (2) a delexicalized version in which the verbal context was obscured digitally by means of a low-pass filter while preserving the intonation, pacing and pitch, and (3) a pitch-only version (where the audio stream was converted digitally into pure tones, while preserving rhythmic content). The delexicalized and pitch-only versions were created from the original audio clips using Praat 6.0.2 software (www.praat.org). Examples are given in the Supplementary Materials*.* Audio files are available on request.

The delexicalized versions were obtained using the low-pass filter by means of the Praat command *Filter* → *Filter (pass Hann band)*, with the following parameters: From Frequency: 0 Hz, To Frequency = 1000 Hz, Smoothing = 100 Hz. The sets of delexicalized clips were independently checked by two other authors (one a native English speaker, the other a native Spanish speaker), who were unable to identify any words clearly. The net effect is to leave the audio clip much as would be experienced if one were sitting in a busy bar or café where the rise and fall of voices and intonation of a nearby conversation could be heard, but not the actual words themselves.

The pitch-only versions were produced in two steps: by first generating a pitch object by means of the Praat command *Analyse Periodicity* → *To pitch*, and then transforming this by means of the Praat command *To sound (sine)*, with a default Sampling Frequency and cutting voiceless stretches at the nearest zero crossings. Thresholds corresponded to 75–300 Hz for male-male conversations, 100–500 Hz female-female ones, and 75–500 Hz for female-male exchanges. Some clips were extracted from compressed YouTube files, the quality of which could obstruct Praat’s pitch extraction algorithm. For this reason, pitch-only files were compared sentence-by-sentence against the original files in order to locate and correct autocorrelation errors (e.g. octave jumps). The rhythmic content of these clips was partially preserved. While losing the signal’s intensity (and therefore some forms of stress within an utterance), the resulting pitch-only version of the clips retained pitch accents, as well as the overall pace of the speech, and the exact temporal placement and duration of both phonation and silences.

Participants first completed background information checks, and then listened to the 24 audio clips (8 relationships in full audio, delexicalized, and as pure tones). Half listened to English clips and half listened to (Chilean) Spanish clips, with participants from the two study populations randomly assigned to each condition. In all four population-by-condition cases, participants heard the clips in the same order: first the eight delexicalized clips, then the eight pitch-only clips, and finally the eight full audio clips, with the order of the eight clips in each case randomized. After listening to each clip, participants were asked to decide which of the eight registers (relationship types) listed in Table 1 best characterized the relationship between the speakers in the clip they had just listened to. The English and Spanish prompts used for the eight registers are given in Table S2 in the Supplementary Materials*.* The eight choices were listed in randomized order on each presentation.

To check whether acoustic features alone might account for some of our results, we assessed the means, modes, and standard deviations of the frequencies (f_0_) of each clip. On Sonic Visualiser (Release 2.5), we used the *Melodia* melody extraction plugin, with the following parameters:

**Table:** 

Program	Polyphonic
Min Frequency	55.00
Max Frequency	1760.00
Voicing Tolerance	0.20
Monophonic noise filter	0.00
Audio frames per block	2048
Window increment	128

We transferred the "Layer data" to Excel, removed negative frequency values (an artifact of the program), and extracted mean frequencies and standard deviations (in Hz). We then expressed standard deviations of the frequency for each clip as a proportion of the equivalent rectangular bandwidth (ERB) at the clip's mean f_0_ (in order to perceptually normalize frequency variability across the clips). ERB for each center frequency was calculated using Glasberg and Moore's (1990) formula:

$$ERB\left( f \right) = 24.7 \times \left( {4.37 \times f + 1} \right)$$

(where $f$ is the center frequency in kHz, and ERB ($f$) is the bandwidth of the auditory filter in Hz). The results are shown separately for the two languages in Table 2. As is evident in the columns headed *SD*_**ERB**_, frequency variability values are higher in English than in Spanish, as are mean and modal f_0_ values (except in one case). This is characteristic of the two languages. In both languages, *radical disagreement* and *malicious provocation* (unambiguously negative) are associated with high mean f_0_ and wide variability in frequency; in general, wide frequency variability is associated with negative rather than positive interactions across both languages, with the exception of *friendly provocation* in English. In both languages, the mean f_0_ and frequency variability for the category *gossip* approximate those of positive interactions, which might lead to some capacity to confuse the two.

**Table 2:** Comparison of acoustic properties in the audio clips for the eight registers

	English			Spanish		
Register	modal f	mean f	*SD*_ERB_	modal f	mean f	*SD*_ERB_
Positive						
Free agreement	160	172	1.02	137	127	0.61
Disagree with respect	166	180	0.98	148	153	0.87
Phatic communion	166	182	1.10	162	142	0.67
Friendly provocation	151	193	1.61	119	115	0.90
Negative						
Enforced agreement	162	186	1.53	165	198	1.14
Radical disagreement	392	356	1.33	260	249	1.30
Malicious gossip	150	178	0.97	198	207	0.81
Aggressive provocation	357	398	1.10	302	306	1.00

## Results

**Fig. 1:**
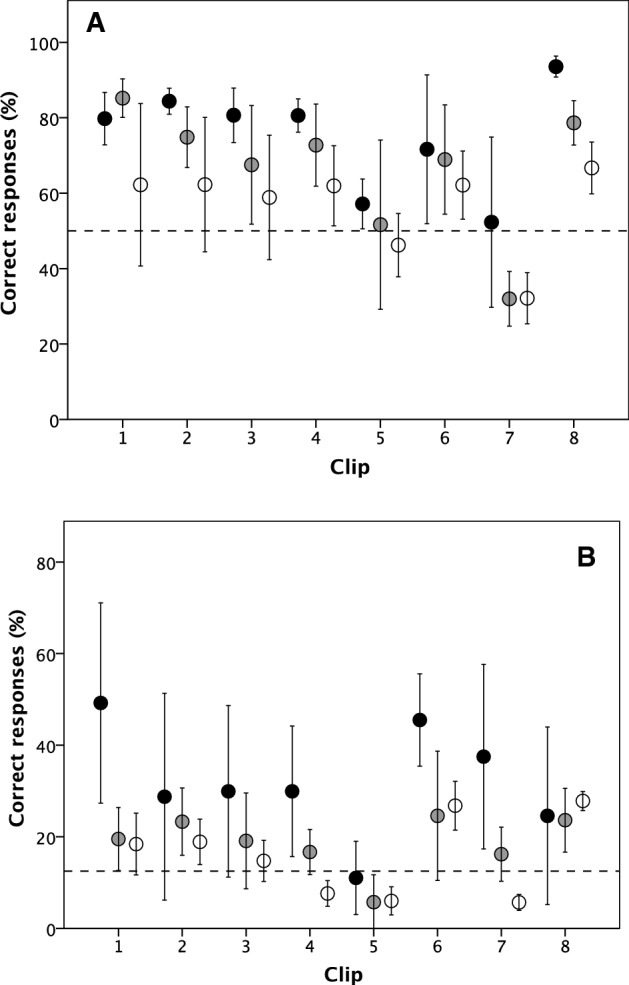
Mean (± 2 se) percent correct responses for the eight relationship types shown in Table 1 for **a** positive versus negative relationships only and **b** actual register. English and Spanish subjects pooled. Filled symbol: full audio; grey: delexicalised; unfilled: pitch only. Dashed line: expected value for random responses (50% for [a] and 12.5% for [b]. Clips 1–4: positive relationships; clips 5–8: negative relationships (for definitions, see Table 1). The data for each clip in each condition are given in Table S1

We first calculated percent correct responses across subjects for the 96 independent cells partitioned by population (UK vs. Spain), clip language (English vs. Spanish), condition (audio vs. delexicalized vs. pitch) and clip (the 8 relationship types). The results for all 96 cells are given in the Supplementary Materials, Table S3. Figure 1 summarizes the results as mean scores across the three conditions (both population and both language samples combined) for the 8 individual clips. The dashed horizontal line indicates random choice (for the binary case, this is 50%; for the full eight registers, this is 12.5% correct).

We analyzed the results at two levels: the more general level used in most previous studies (did participants correctly identify the relationship as positive or negative, with a sample of four clips of each kind), and then a more detailed one (did participants correctly identify each of the eight registers, or relationship types, correctly, with just one clip per register).

For binary identification (positive versus negative only), mean performance approximated random expectation only for audio clip 5 (enforced agreement) and clip 7 (malicious gossip) (Fig. 1a). All the other means were significantly above random. This was broadly the same for all eight registers separately, although subjects generally did worst on clip 5 (Fig. 1b). A one sample test for each of the three conditions comparing against an expected value of 50% correct on a binary positive/negative choice and a 12.5% correct for identifying the exact relationship register yields significantly higher accuracy than would be expected by chance for all three conditions on both outcome variables (binomial tests, 1-tailed for a directional hypothesis; binary ratings: full audio, *p* = 0.004; delexicalized, *p* = 0.004; pitch only, *p* = 0.026; exact relationship: full audio, *p* = 0.016; delexicalized, *p* = 0.016; pitch only, *p* = 0.001). Though performance is far from perfect, subjects clearly did better with nonverbal cues than might be expected if they responded at random. Compared to full audio, delexicalized is 88.6% as accurate on binary ratings and 53% on individual relationship registers (against expected values of 50% and 12.5%, respectively), while pitch-only is 75.4% and 44.9% as accurate, respectively. These results support hypothesis H1b over H1a: while verbal cues clearly add significant information, nonverbal cues on their own clearly allow better than chance performance.

Figre 1 (and Supplemnary Materials, Table S3) confirm that, broadly speaking, performance on pitch-only samples is poorer than on delexicalized samples (there is much less information available): performance ranks in the order full audio > delexicalized > pitch-only. Hypothesis H2b is, thus, favored over H2a, suggesting that the range of information content in the cues available is important.

**Fig. 2:**
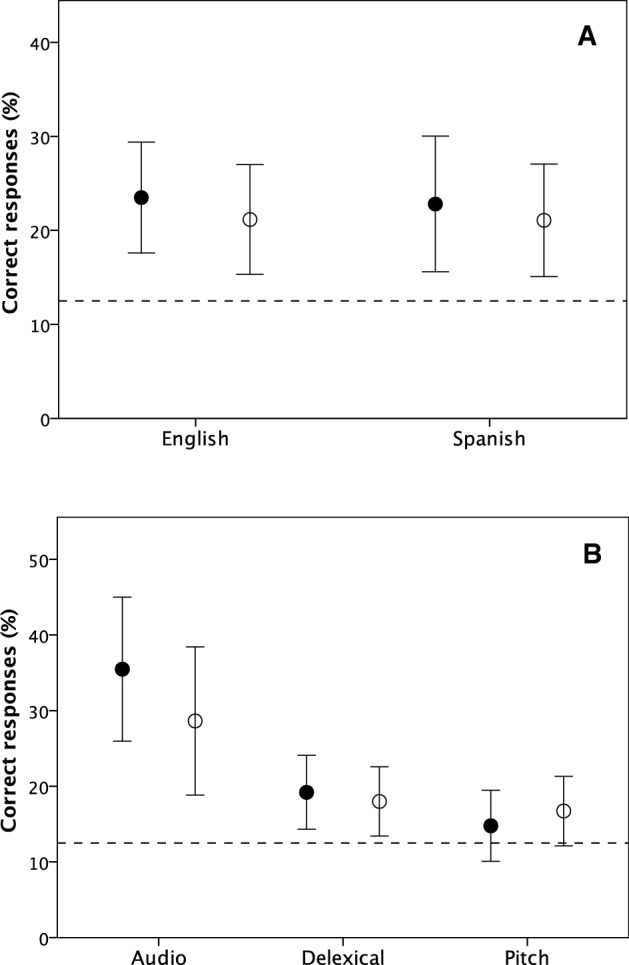
Mean (± 2 se) correct response rates for English (unfilled symbols) and Spanish (filled symbols) speakers in identifying correct register **a** for the 8 clips in their own and the other language and **b** the clips in both languages combined in the three conditions. In each, case, responses are pooled across all other conditions

There were no differences in performance between Spanish and English speakers when listening to their own or the other language (Fig. 2a). Nor was there a significant difference between the two populations in their performance on the different types of clip (Fig. 2b). On average, participants correctly identified the relationship as positive or negative on ~ 35% of audio clips, ~ 19% of delexicalized clips and 16% of pitch-only clips (Figre 2b). Both populations experienced most difficulty with enforced agreement (the register we, in fact, had most difficulty finding samples for). Not surprisingly, both populations did much better on the full audio clip than on the delexicalized version or the pitch-only versions (Fig. 2b). Hypothesis H3 is not supported.

A GLM with percent correctly identified as positive or negative relationship as the dependent variable, clip format as a fixed factor and nationality, register and clip language as random factors yields a significant overall model (*F*_11, 84_ = 13.81, *p* < 0.0001). Neither nationality (*F*_1, 84_ = 1.14, *p* = 0.289, η^2^ = 0.013) nor language (*F*_1, 84_ = 0.69, p = 0.408, η^2^ = 0.008) had significant effects on performance, but both format (*F*_2, 84_ = 18.03, *p* < 0.0001, η^2^ = 0.300) and clip register (*F*_7, 84_ = 19.09, *p* < 0.0001, η^2^ = 0.614) did so.

A GLM analysis with percent actual relationship register correctly identified also yields a significant model (*F*_5, 90_ = 5.46, *p* < 0.0001). Language (*F*_1, 84_ = 1.17, *p* = 0.283, η^2^ = 0.014) was, again, not significant (Fig. 2a), but there were significant effects due to nationality (*F*_1, 84_ = 6.20, *p* = 0.015, η^2^ = 0.069), format (F_2, 84_ = 13.39, *p* < 0.0001, η^2^ = 0.242) and clip register (*F*_7, 84_ = 5.19, *p* < 0.0001, η^2^ = 0.302). Although native Spanish speakers typically performed better than native English speakers, the effect size (η^2^ = 0.069) was very modest compared those for format (η^2 k^ = 0.242) or clip register (η^2^ = 0.302).

We checked for five possible sources of confound in our results: variation in f_0_, fluency in second language, length of clip, imbalance in the sex ratio of subjects and the use of clips from reality shows.

Although most f_0_ modal values and variances were similar, there were some differences in the clips used both within and between languages. To determine whether this might have biased the results, we ran an analysis of variance with percent correct responses as the dependent variable and f_0_ and sd_ERB_ as the independent variables, for the 32 clip type, subject population and language combinations for the full audio sample. The overall model is not significant (*F*_15,16_ = 1.95, *p* = 0.099), and neither of the independent variables have significant individual effects on percent correct responses (f_0_: *F*_1, 16_ = 1.30, *p* = 0.272, η^2^ = 0.075; sd_ERB_: *F*_2, 16_ = 2.21, *p* = 0.143, η^2^ = 0.216).

**Fig. 3:**
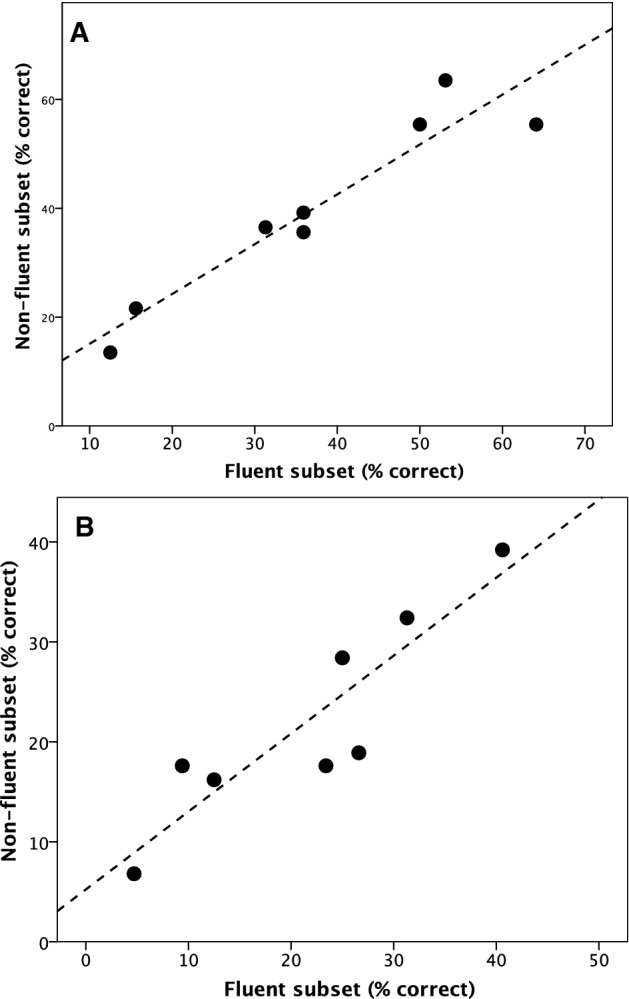
Performance on **a** full audio and **b** delexicalized English clips for Spanish subjects who were fluent in English compared to those who were not fluent. Dashed line is least-squares regression

To check whether fluency in the second language makes any difference to the ability to identify interaction type, we compared fluent with non-fluent second language speakers. Because too few subjects in the English sample declared any degree of fluency at all in Spanish, this analysis could only be carried out for the Spanish sample. We compared performance by the two subsets on the delexicalized and full audio English language clips (Fig. 3). In both cases, performance on identifying which of eight possible relationship types was involved was highly correlated between the two fluency subsets (full audio: *r* = 0.949, *N* = 8, *p* < 0.0001; delexicalized: *r* = 0.901, *p* = 0.0005; 1-tailed in each case since we test a directional hypothesis [a significant negative correlation would obviously not be evidence *for* the hypothesis]). In neither case does the regression slope differ from *r* = 1 (*t*_6_ = 0.68, *p* = 0.522; *t*_6_ = 1.44, *p* = 0.200 2-tailed). Both groups experienced the same level of difficulty with the same clips (clips 5 and 8 in the full audio condition, and clips 1 and 5 in the delexicalized condition). As might be expected, the correlation for the delexicalized clips is poorer than that for the full audio clips.

Although the clips presented as stimuli were of broadly similar length (all were short), they did range in length between 11–64 s, and this might have been sufficient to bias the results (longer clips might give more time in which to decipher the type of relationship). To check whether this might explain our results, we correlated percentage correct answers (correctly identified relationship type) with clip duration. We used only the full audio clips for this so as to maximise chances of correctly inferring the right answer. In non of the four language combinations does clip duration correlate significantly with response accuracy (Supplementary Materials Figure S1; English-English: *r* = -0.048, *p* = 0.911; English–Spanish: *r* = 0.347, *p* = 0.399; Spanish–English: *r* = 0.563, *p* = 0.146; Spanish-Spanish: *r* = -0.635, *p* = 0.091). In other words, within the range of clip durations we used, clip length does not influence capacity to correctly categorize the interaction type.

We did not include gender as a variable in our analyses, since it is not customary to do. However, since there was a slight imbalance in the sex ratio of subjects in favour of females (65% female in the English sample, 60% female in the Spanish sample) whereas the reverse was the case for the stimuli clips (60% males), it is conceivable that this might have biased the results if one sex performed better than the other or same-sex cues were easier to interpret than opposite-sex cues. We checked this by comparing male and female success in identifying the eight relationship types in the four experimental conditions on the full audio clips. In fact, scores for the two sexes are highly correlated individually and overall (Fig. S2; pooled sample: *r* = 0.842, *N* = 32, *p* < 0.0001; English hearing English: *r* = 0.902, *N* = 8, *p* = 0.002; English hearing Spanish: *r* = 0.949, *N* = 8, *p* < 0.001; Spanish hearing English: *r* = 0.672, *N* = 8, *p* = 0.068; Spanish hearing Spanish: *r* = 0.920, *N* = 8, *p* = 0.001).

Finally, we checked whether the use of reality TV show material influenced the results. There was only one clip of this kind in the Spanish dataset, but half the English clips were from reality shows (Supplemenary Materials, Table S1). We compared responses to clips that were reality show material versus those that were not for the two nationalities separately on the full audio and on the delexicalized versions. Three tests were non-significant, with only the English subjects' responses on the delexicalized versions being just significantly different – and then, contrary to prediction, they actually did worse on the reality TV clips (English audio: *F*_1, 6_ = 0.04, *p* = 0.853; English delexicalized: *F*_1, 6_ = 6.30, *p* = 0.046; Spanish audio: *F*_1, 6_ = 0.06, *p* = 0.817; Spanish delexicalized: *F*_1, 6_ = 0.05, *p* = 0.835).

## Discussion

Our study aimed to test whether it is possible to infer the quality of an interaction or relationship between third parties simply from the auditory cues contained in their conversation. Whereas most previous studies focus on the cues provided by simple utterances (how do we recognize the emotional content in an utterance), we sought to go behind the simple broadcasting of signals to examine the way sets of these cues concatenate in a conversational sequence and allow us to read the quality of the relationship in an interaction—the rapport between speakers, the history of their relationship. We compared responses by the native speakers of two European languages to their own and the other language when listening to short excerpts of natural conversation. Subjects were able to identify the quality of the relationship, both in terms of a simple positive/negative dichotomy and in terms of finer sub-categories within these, at significantly better than chance levels simply from the auditory cues alone, shorn of their verbal content. Moreover, they were able to do so even when the utterances were stripped of their verbal (but not prosodic) content.

The results confirm (1) that the nonverbal cues of conversational exchanges alone provide significant information about the quality of a relationship (its register, or “rapport”) and (2) that, nonetheless, there is an incremental benefit from having enhanced prosodic richness and more explicit verbal information. Hypothesis H1b was supported over H1a (performance was significantly higher than chance in all three conditions), and H2b over H2a (performance ranked in order of degraded information). Finally, hypothesis H3 (that performance would be poorer on an unfamiliar language) was rejected, indicating that sufficient (nonverbal) cues remain available to correctly identify relationship quality even in an unfamiliar language. An important feature of our design is that we asked subjects to identify the quality of the relationship between two people engaged in a conversation based on a short clip extracted from the conversation.

Overall, the lowest rates of correct response were on enforced agreement and malicious gossip (clips 5 and 7: Fig. 1a), both of these being negative registers (the difference between these and the other six registers was significant: *F*_1, 30_ = 10.96, *p* = 0.003, η^2^ = 0.274). This may be because a wider range of cues (e.g., visual cues) are needed to clarify the meaning or significance of the interaction in these cases or because the criteria offered were insufficiently precise (though we doubt this, given that there is no interaction effect of population, own versus other language or register on percent correct responses: *F*_4, 24_ = 1.50, *p* = 0.234, η^2^ = 0.200).

The difficulty subjects experienced in classifying all versions of clip-5 (enforced agreement: one speaker does not really agree with the other but feels obliged to do so, out of politeness or deference) may be related to the fact subjects were much more likely to misclassify the full audio version as the adjacent closely related register (disrespectful disagreement). This was equally true for subjects listening to clips in their own language and the other language. We established that these results were unlikely to be explained by second language proficiency, clip length, or participant gender. Some subjects also found the delexicalized and pitch-only versions of clip-4 (friendly provocation: speakers winding each other up in a friendly way) and clip-7 (malicious gossip) difficult to classify. There was a tendency to misclassify friendly provocation (teasing, banter) as friendly agreement (certainly a reasonable alternative) and malicious gossip as phatic communion (arguably, a plausible confusion when there is no clegar knowledge as to who the referent is or visual cues to offer enlightenment), both of which signal a positive interaction.

On the delexicalized clips, participants performed, on average, at 80% the accuracy of the full audio clips for binary decisions (positive versus negative affect, as in most previous studies). In other words, verbal (semantic) content seems to provide only an additional 20% information. Mehrabian (1972) had claimed that 55% of the information in speech came from visual signals (facial expressions and gestures), with just 7% from verbal content. If we exclude the visual component from Mehrabian’s estimates, the 7% he claimed was due to verbal content would equate to 7/(100–55) ≈15% of the non-visual content of an interaction. This is close enough to the 20% that we found to suggest that, his critics notwithstanding, he might have been broadly right, at least in the context of social exchanges.

The fact that English and Spanish speakers were as accurate on each other’s languages as on their own (Figs. 1 and 3) reinforces this claim. It suggests that semantic information may not be completely necessary for understanding the quality of relationships among strangers, even though the verbal content may add significant additional information. It also suggests that prosodic cues may be culturally quite similar, at least across Indo-European languages, despite marked cultural differences in both speaking style (e.g., delivery speed, intonation) and expression. English makes use of wider variations in pitch than Spanish does (Farías, 2013), but these additional aspects of cultural “style” do not seem to make a significant additional contribution.

These results support Grahe and Bernieri’s (1999) findings that nonverbal and/or visual cues are often sufficient to establish the presence of rapport between two speakers. They also confirm the findings of Cowen et al. (2019) that prosodic cues alone are sufficient to allow individuals to identify the emotional quality of an utterance. In both the latter cases, speakers were either strangers cooperating on a task or professional actors reading scripts in different emotional tones, whereas in our case individuals typically knew each other and had an established relationship. Importantly, our study extends their findings to natural conversations, demonstrating that the earlier findings really do generalize to natural situations outside the laboratory.

Although we are not here concerned to identify the particular acoustic features that underlie the capacity to recognize differences in relationship quality, it is worth noting that previous work has identified a number of plausible candidates. Fundamental frequency (f_0_), for example, may be of central importance in shaping prosodic judgments in a wide range of different languages and contexts. Yaeger-Dror (2002) explored a range of comparable interactive registers in French and American English and found that "variation in fundamental frequency appears to be the primary realization of focal prominence" (p. 1497). Greenberg et al. (2009) analyzed single utterances of "n" in samples of native-born Japanese in conversation (in Japanese, “n” can be an interjection, a rejoinder, or a filler, depending on the context) and found that these could be consistently differentiated in terms of their sociolinguistic functions on the basis of f_0_ height and dynamic pattern, with high f_0_ correlating with positive judgments and low f_0_ with negative. Similarly, in a recent series of experiments, Hellbernd and Sammler (2016) found that single words produced so as to indicate a set of different speech acts (or registers: criticism, doubt, naming, suggestion, warning, and wish) could reliably be sorted into the appropriate speech act categories on the basis of acoustic features (including mean f_0_). They found that listeners could reliably categorize the words used as stimuli, and that these categorizations could be predicted by the specific acoustic features. Similar features may presumably be expected to explain some of our findings, although our study was not intended to examine this question. Although there were modest differences in f_0_ between registers in our study stimuli, these did not predict how well subjects identified the different kinds of rapport characteristic of the relationship depicted. This is not, of course, to say that other aspects of speech production may not be important.

There are two issues with our methodology that would benefit by further consideration. One concerns the selection of clips for use in the experiments. The other issue concerns extraneous variability in the quality of the clips that might result in confounds in the way subjects classified them.

We used the concept of registers to provide a framework for spreading the choice of audio clips across a wide range of possible relationships and, more importantly, to ensure that the clips for the two languages were as closely matched as possible in this respect. Our concern was not to test the validity of registers as a concept, but simply to ensure that the clips did not end up being too similar to each other, or too different between the two languages. Choice of clips was then at the discretion of the two authors who made the selections, subject to their attempts as far as possible to match their choices for the two languages. Their choices were subsequently audited blind by other members of the research team. Future studies might consider a wider range of relationship types and a greater variety of examples within each type.

A second issue relates to the fact that we used audio clips recorded under natural conditions rather than in the laboratory. This could result in marked differences between clips in sound intensity, as well as other contextual cues. The latter, of course, is why we did not use the full video clips. Although we did not attempt to control for sound intensity, there are four reasons for thinking that this is unlikely to have been a problem. First, the samples for the two languages were recorded under very different conditions, yet performance on the two language samples are highly concordant for both populations of subjects. Second, if only sound intensity explains the results, then subjects’ responses should be random since there is no reason why sound intensity should be correlated with register. The results are self-evidently not random. Third, all four positive clips in the Spanish set were in fact recorded under laboratory conditions (see Supplementary Materials, Table S1); nonetheless, responses vary across the four samples in a way that is very similar to that observed in the equivalent English clips (all of which were recorded in high quality broadcast sound: see Supplementary Materials, Table S1). Fourth, sound intensity was unavoidably altered by the software (in producing the delexicalized clips) and directly removed (in the pitch-only versions), and despite this subjects managed to differentiate registers in a similar pattern to the full audio and were able to do so equally well on audio clips recorded under two very different conditions in the two language samples.

One final issue to consider is the implications that our findings have for understanding online communication media. The substantive point to emerge from an extensive literature on this topic is that the availability of nonverbal cues significantly enhance the perceived quality of interactions not just between existing friends but also between strangers (Finkel et al., 2012; Frost et al., 2008; Kotlyar & Ariely, 2013; Vlahovic et al., 2012; Whitty, 2008). There is a natural progression from text-based media (email, social networking sites) through aural media (phone, radio) to video-embedded media (skype, zoom) that enables progressive layers of information richness (verbal, verbal plus auditory, verbal plus auditory plus visual, respectively). Each step greatly enhances the inferences that can be made (Finkel et al., 2012; Hegstrom, 1979), and hence the satisfaction gained from an interaction (Kumar & Epley, 2021; Schroeder et al., 2017; Vlahovic et al., 2012).

However, even video-embedded digital media leave something to be desired in terms of the sense of satisfaction that interactions give us (Nadler, 2020; Vlahovic et al., 2012). In part this is due to a combination of factors, including a screen-size limitation on the number of people that can be comfortably accommodated visually, the prominence of distracting backgrounds, difficulty in making direct eye contact and in identifying who is speaking (i.e., lack of aural triangulation), lack of intimacy and privacy, disruptive fading or image jitter, the intrusion of extraneous noise due to omnidirectional microphones, the temporal unpredictability of the audio signal introduced by the buffering typical of unstable internet connections, and the fact that conversations are necessarily limited to a single speaker irrespective of how many people are in the call (a constraint imposed by the dynamics of everyday conversations: Dahmardeh & Dunbar, 2017; Dunbar, 2016; Dunbar et al., 1995; Robertson et al., 2017). While these environments certainly work well for one-on-one (or even one-on-two) interactions, their functionality does not yet extend to social groupings (other than those in lecture format) or, perhaps, to casual observation of third parties of the kind that we focus on here.

## Supplementary Information

Below is the link to the electronic supplementary material.

Supplementary file1 (DOCX 2676 kb)

Supplementary file2 (MP3 1784 kb)

Supplementary file3 (WAV 7842 kb)

Supplementary file4 (WAV 3921 kb)

Supplementary file5 (XLSX 29 kb)

Supplementary file6 (XLSX 24 kb)

Supplementary file7 (XLSX 11 kb)

## Data Availability

The data are available at https://doi.org/10.5287/bodleian:NydVzojaq.
