# Supplementary material for: Nonverbal Auditory Cues Allow Relationship Quality to be Inferred During Conversations
Source: J Nonverbal Behav. 2021 Oct 22;46(1):1–18. doi: 10.1007/s10919-021-00386-y (PMC8881250; doi:10.1007/s10919-021-00386-y)
Supplement: Supplementary file 1 — Supplementary file1 (DOCX 2676 kb) [file 10919_2021_386_MOESM1_ESM.docx]

**Supplementary Methods**

**Table S1. Sources for audio clips**

Relationship Sources

type English speakers‡ Spanish speakers‡

**Positive interactions:**

1. Free agreement *Life of Riley* vlog* MF LIF project conversation ^¶^ MM

2. Respectful difference of opinion *Life of Riley* vlog* MF LIF project conversation ^¶^ MM

3. Phatic communion *Life of Riley* vlog* MF LIF project conversation ^¶^ MM

4. Friendly provocation: *Life of Riley* vlog* MF LIF project conversation ^¶^ MM

**Negative interactions:**

5. Enforced agreement *I’m a Celebrity UK*† FF *Tolerencia 0* TV panel discussion^§^ MM

6. Disrespectful difference of opinion *Big Brother UK*† FF *Tolerencia 0* TV panel discussion^§^ MF

7. Malicious gossip *Big Brother USA*† MM(F) *La Granja* TV reality show MF

8. Aggressive provocation *Big Brother UK*† MF Universidad del Mar FF

recorded discussion

‡ M = males; F = females (all adults); in some cases, other people are present on-screen but do not contribute to the conversation, aside from a brief comment in one case (indicated by (F) in clip 7)

* a regular vlog by a young married couple

^¶^ Language Interaction and Phenomenology Lab (LIF) research project in collaboration with the Cambridge University

Centre for Music and Science; excerpts from same recording series involving the same two people

† Reality TV shows

§ Chilevision TV regular weekly panel discussion programme

All clips were originally video clips. Only the audio tracks are used in this study.

**Table S2. Prompts for the 8 registers (relationship types)**

The 8 registers are intended simply to provide a way of ensuring that the clips represent a range of interaction types, rather than all being of a single type (happy, aggressive, etc). We are not here concerned to justify the registers, but simply regard them as a useful, commonsense way of dividing up the range of different interaction qualities than can occur in real life. They need not even be an exclusive division of the state space of interaction types. Our main concern is simply to try to have some variety.

After listening to each clip, subjects were asked to classify the relationship between the speakers as one of the following eight (presented as 8 consecutive statements without separating them into positive vs negative). For each clip, the order of the eight statements was randomised.

1. **English**

***Positive*:**

1. The speakers are in agreement with one another
2. The speakers have different opinions to one another but wish to retain a positive

relationship

1. The speakers are not really bothered about the topic of conversation but are just

passing time together

1. The speakers are winding each other up as a joke but do not intend to upset each other

***Negative*:**

1. One speaker says they agree but they are lying – what they are saying is different to

what they think

1. The speakers have a difference of opinion and do not care if they upset / insult the

other

1. The speakers are talking maliciously about someone who is not present
2. One speaker is intentionally and maliciously trying to upset the other person
3. **Spanish:**

***Positive*:**

1. Los interlocutores están de acuerdo entre sí
2. Los interlocutores tienen opiniones diferentes entre sí, pero desean mantener una buena relación
3. Los interlocutores no están realmente preocupados por el tema de conversación, sino que sólo están pasando tiempo juntos
4. Los interlocutores se están tomando el pelo el uno al otro en broma, pero no tienen intención de molestarse

***Negative*:**
5) Un interlocutor dice que están de acuerdo, pero mienten: lo que dicen es diferente

de lo que piensan.

1. Los interlocutores tiene diferencias de opinión y no les importa si molestan o

insultan al otro

1. Los interlocutores hablan maliciosamente de alguien que no está presente
2. Uno de los interlocutores está tratando de molestar a la otra persona de manera

intencional y maliciosa

**Tests of Potential Confounds**


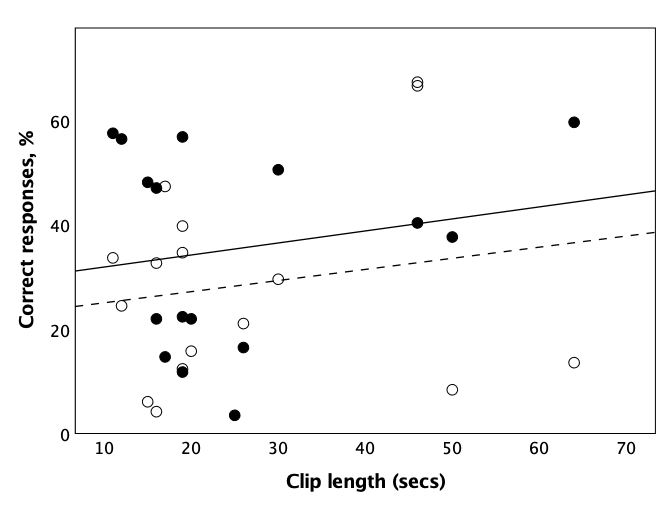


*Figure S1*

*Mean percent correct responses on the 8 full audio clips as a function of clip duration (all subjects pooled). Symbols: filled, Spanish speakers; unfilled, English speakers.*

*
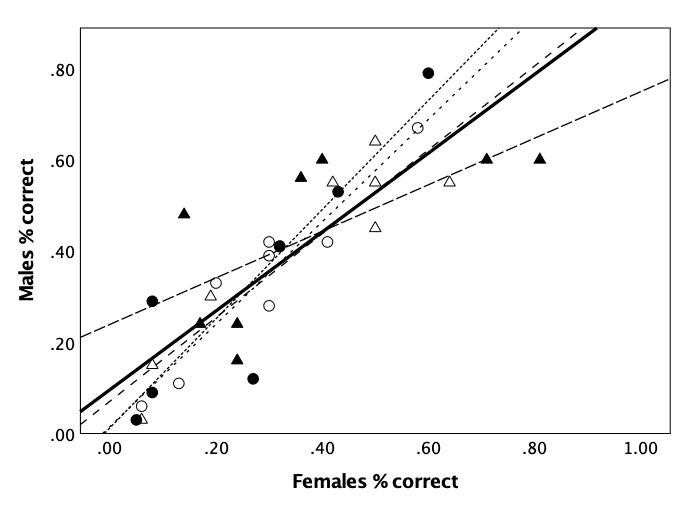
*

*Figure S2*

*Percent correct on each of the 8 audio clips by male subjects plotted against the equivalent values for females. Symbols: circles, English-speakers; triangles, Spanish speakers; filled, hearing English clips; unfilled, hearing Spanish clips.*
